# The pivotal roles of gut microbiota in insect plant interactions for sustainable pest management

**DOI:** 10.1038/s41522-023-00435-y

**Published:** 2023-09-21

**Authors:** Yuxin Zhang, Shouke Zhang, Letian Xu

**Affiliations:** 1 State Key Laboratory of Biocatalysis and Enzyme Engineering, School of Life Sciences, Hubei University, 430062 Wuhan, China; 2 State Key Laboratory of Subtropical Silviculture, Zhejiang A & F University, 311300 Hangzhou, China

**Keywords:** Symbiosis, Microbiome

## Abstract

The gut microbiota serves as a critical “organ” in the life cycle of animals, particularly in the intricate interplay between herbivorous pests and plants. This review summarizes the pivotal functions of the gut microbiota in mediating the insect–plant interactions, encompassing their influence on host insects, modulation of plant physiology, and regulation of the third trophic level species within the ecological network. Given these significant functions, it is plausible to harness these interactions and their underlying mechanisms to develop novel eco-friendly pest control strategies. In this context, we also outline some emerging pest control methods based on the intestinal microbiota or bacteria-mediated interactions, such as symbiont-mediated RNAi and paratransgenesis, albeit these are still in their nascent stages and confront numerous challenges. Overall, both opportunities and challenges coexist in the exploration of the intestinal microbiota-mediated interactions between insect pests and plants, which will not only enrich the fundamental knowledge of plant–insect interactions but also facilitate the development of sustainable pest control strategies.

## Introduction

Arthropoda is one of the most diverse and successful animal phyla, boasting an estimated 5.5 million insect species, of which only one million species have been named thus far^1^. More than half of these species are herbivorous insects, which have caused significant economic losses and serious ecological problems to agricultural and forestry production. It has been reported that global crop production suffers a loss of more than 15% annually due to these pests^2^. In recent years, the issue of pests has been compounded by factors such as climate change, rapid globalization, and urbanization, as well as the spread of invasive species^3,4^. While chemical pesticides have been widely employed to combat pests, their injudicious use has led to various problems, including the disruption of natural ecosystems, the emergence of pesticide-resistant pests, and adverse health effects on humans^5–7^. Hence, it is crucial to identify eco-friendly methods for pest control in agriculture and forestry.

Insects harbor a diverse and abundant microbial population in their gut system^8^. Some gut bacteria play a positive role in adapting pests to host plants, including providing nutrition, aiding digestion, detoxification, and directing pest behavior^9–11^. The symbiotic relationships between gut bacteria and insect pests have partly contributed to the success and diversification of insects, but have also increased the difficulty of pest control. Interestingly, some studies have indicated that the function of gut bacteria can change and become detrimental to the host insects when a third species participates in the pest–plant interaction or due to other abiotic factors^12–14^. Therefore, comprehensively understanding the function of gut bacteria in multispecies cascading interactions can not only unveil novel resources for biocontrol but also facilitate the development of new biopesticides.

Many reviews have summarized the functions of gut bacteria in insects, but there have been few reviews that have highlighted the diversified functions of gut bacteria in the context of insect–plant interactions, especially in multispecies cascading interactions^7,9,15–17^. Drawing on previous studies, we will describe the role of gut bacteria in this system from multiple perspectives, and discuss how to screen key bacteria and apply these theories to pest control in combination with other techniques.

## Gut bacteria affect the insect–plant relationship by affecting the host insect

**Fig. 1:**
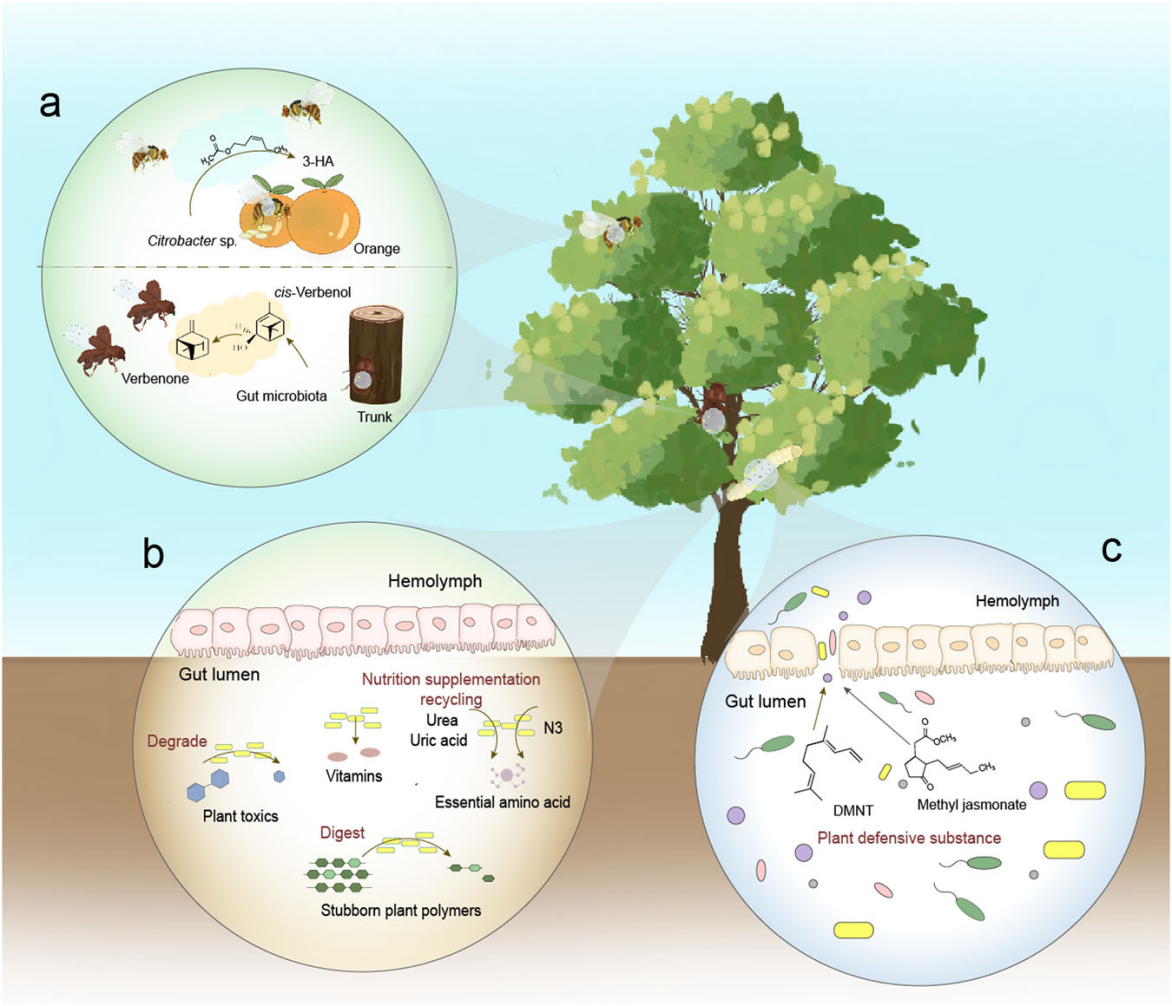
Known functions of insect gut bacteria that directly influence insect behavior and physiology. **a** Gut bacteria mediate insects’ plant selection preference; **b** Gut bacteria assist insects in overcoming feeding obstacles; **c** Plant defense-induced dysbiosis of gut microbiota is lethal to insects.

Herbivorous insects have adapted to plants that provide heterogeneous resources, which can be considered as the “center of activity” for the insects^18^. These adaptations may be facilitated by the direct and indirect interactions of insect gut bacteria with host plants^19^. Among these interactions, certain gut bacteria possess ecological functions that directly influence insect behavior and physiology, which can be generally categorized as follows (Table 1 and Fig. 1); firstly, gut bacteria have been shown to mediate the plant selection preference of insects, assisting them in finding suitable host plants for survival and reproduction (Fig. 1a). Once herbivorous insects successfully locate and attack the selected plants, they often encounter substantial challenges in feeding, including low nutrient content, indigestibility, and toxicity of many plant tissues^11^. Some insect gut bacteria confer the ability to overcome these feeding obstacles, allowing insects to develop and reproduce on plants (Fig. 1b). Conversely, chemicals or inadequate nutrition in plant tissue cause dysbiosis in the gut microbiota of herbivores insects, which often have adverse effects on their biology (Fig. 1c).

**Table 1:** Gut bacteria participate in plant–insect interactions by influencing insect biology.

Insect	Order	Gut bacteria	Bacteria function	References
**1. Gut bacteria mediate host plant preferences of insects**				
*Frankliniella occidentalis*	Thysanoptera	*Erwinia* sp.	Attracting larvae	^24^
*Dendroctonus valens*	Coleoptera	Gut microbiota	Assisting beetles to determine the suitability of the pine tree for colonization	^25,26^
*Bactrocera dorsalis*	Diptera	*Citrobacter* sp.	Attracting female flies to lay eggs on the host fruit by VOCs	^10^
**2. Gut bacteria digest stubborn plant polymers or providing nutrients that plant lacks**				
*Macrotermes gilvus*	Isoptera	*Provedencia sp*. *Bacillus sp*.	Production of cellulase	^31^
*Sirex noctilio*	Hymenoptera	*Streptomyces* *Pantoea*	Production of cellulase	^29^
*Cyrtotrachelus buqueti*	Coleoptera	*Bacillus velezensis*	Production of cellulase	^146^
*Osphranteria* *coerulescens*	Coleoptera	*Bacillus safensis*	Production of cellulase	^147^
*Holotrichia* *parallela*	Coleoptera	*Pseudomonas* sp.	Production of cellulase	^148^
*Cossus cossus*	Lepidoptera	*Bacillus circulans*	Production of cellulase	^33^
*Diatraea saccharalis*	Lepidoptera	*Bacillus pumilus* *Klebsiella oxytoca*	Production of cellulase	^32^
*Cryptotermes brevis*	Isoptera	*Bacillus* sp.	Production of ligninase	^42^
*Odontotermes obesu*	Isoptera	*Trabulsiella* sp.	Production of ligninase	^41^
*Reticulitermes chinensis*	Isoptera	*Enterobacter hormaechei* *Bacillus licheniformis*	Production of ligninase	^40^
*Hypomeces squamosus*	Coleoptera	*Enterobacter hormaechei*	For feeding and females for egg laying	^149^
*Cassida rubiginosa*	Coleoptera	*Stammera*	Production of pectinase	^36^
*Sphenophorus levis*	Coleoptera	Gut microbiota	Production of pectinase	^150^
*Ceratitis capitata*	Diptera	Enterobacteriaceae sp.	Biological nitrogen fixation	^47^
*Odontotaenius disjunctus*	Coleoptera	*Bacteroidetes* sp.	Biological nitrogen fixation	^52^
*Bactrocera dorsalis*	Diptera	*Morganella morganii* *Klebsiella oxytoca*	Hydrolyzing nitrogenous waste and providing metabolizable nitrogen	^49^
*Melolontha hippocastani*	Coleoptera	*Burkholderia* sp.	Recycling of nitrogen in larvae	^151^
*Riptortus pedestris*	Hemiptera	*Burkholderia* sp.	Supplementation of essential amino acid and B vitamin	^52^
*Dolichoderus* sp.	Hymenoptera	*Bartonellaceae*	Involved in several vitamins and all essential amino acid biosynthetic pathways	^53^
*Dysdercus fasciatus*	Hemiptera	*Coriobacterium glomerans*	Supplementation of B vitamins	^152^
**3. Gut bacteria degrade plant toxins**				
*Hylobius abietis*	Coleoptera	*Enterobacteriaceae* sp.	Degradation of terpenoids	^153^
*Curculio chinensis*	Coleoptera	*Acinetobacter* sp.	Degradation of tea saponin	^154^
*Hypothenemus hampei*	Coleoptera	*Pseudomonas fulva*	Degradation of caffeine	^60^
*Dendroctonus valens*	Coleoptera	*Novosphingobium* sp.	Degradation of phenolic naringenin	^155^
*Psylliodes chrysocephala*	Coleoptera	*Pantoea* sp.	Degradation of isothiocyanates	^156^
*Acrobasis nuxvorella*	Coleoptera	*Bacillus pumilus*	Degradation of *Carya illinoinensis* tannins	^157^
*Thitarodes xiaojinensis*	Lepidoptera	*Raoultella terrigena*	Degradation of quercetin	^119^
*Trichoplusia ni*	Lepidoptera	*Agrobacterium* sp. *Rhizobium* sp.	Degradation of alkaloids	^158^
*Phthorimaea operculella*	Lepidoptera	*Glutamicibacter halophytocola*	Degradation of steroidal glycoalkaloids	^159^
**4. Dysbiosis of gut microbiota is detrimental to insect survival**				
*Plutella xylostella*	Lepidoptera	Midgut microbiota	Assisting plant toxins to kill insects	^13^
*Spodoptera frugiperda*	Lepidoptera	*Enterococcus* sp. *Klebsiella* sp.	Interacting with plant defenses to kill insects	^70^
*Helicoverpa zea*	Lepidoptera	*Serratia marcescens*	Killing insects in synergy with plant defense	^160^
*Spodoptera littoralis*	Lepidoptera	Gut microbiota	Dysbiosis of gut microbiota is detrimental to larval survival	^71^

### Gut bacteria mediate host plant preferences of insects

Host plant preference enables herbivorous insects to choose a suitable host in terms of the fitness of themselves and their descendants^20^, primarily relying on their chemosensory system to analyze different plant volatile organic compounds (VOCs) and make behavioral decisions^20,21^. Indeed, this selection preference is often influenced by microorganisms. While fungi, endophytes, and plant pathogens have been shown to influence insect selection preferences for host plants, a small group of gut bacteria also has a similar effect^22,23^. For instance, western flower thrips prefer thrips-damaged leaves over fresh leaves, which have more gut bacteria on the leaf surface^24^.

Gut bacteria are first transferred to host plants by insects through foraging, excretion, or laying eggs. Once inside the plant, gut bacteria use plant nutrients to spread throughout the plant and produce VOCs that attract or repel intraspecific individuals. In one study, the gut bacteria (*Citrobacter* sp.) of the oriental fruit fly *Bactrocera dorsalis* attracted other female flies to lay eggs on the host fruit by producing 3-hexenyl acetate (3-HA)^10^. The gut bacteria of the bark beetle *Dendroctonus valens* can produce a multifunctional pheromone, verbenone, in its frass, which assists subsequent beetles in determining the suitability of the pine tree for colonization^25,26^. Gut bacteria produce VOCs not only for behavioral attraction but also to highlight the mutual benefit of interacting organisms. While insects can find suitable places to lay their eggs, their gut bacteria have a means of spreading. Although there have been relatively few reports of gut bacteria directly regulating host plant selection, given that this strategy benefits both insects and gut bacteria, we believe this phenomenon will be uncovered in more insect species.

### Gut bacteria digest stubborn plant polymers or provide nutrients lacking in plants

Herbivorous insects tend to feed on all parts of the plant, but digesting stubborn plant polymers presents a challenge as most insects lack the ability to synthesize the necessary enzymes, such as cellulase, pectinase, ligninase, to effectively breakdown these polymers^27^. The gut microbiome has been shown to aid insects’ digestion by enhancing digestive proficiency via enzymatic potentiality. Cellulose is an abundant source of carbon, but it is not readily available to the insect as it exists in the form of crystalline or amorphous microfibers in plant cell walls^28^. It needs to be hydrolyzed into small molecules of sugar by cellulase, a process in which gut bacteria are typically involved^9^. Researchers have identified *Streptomyces* and *Pantoea* in the gut of an invasive wood-eating wasp (*Sirex noctilio*) that provides cellulase to the host insect, enabling the breakdown of cellulose and facilitating nutrient acquisition^29^. Similar functional gut bacteria (e.g., *Provedencia* sp., *Bacillus* spp., and *Klebsiella* spp.) are also found in Blattodea (*Macrotermes gilvus*), Coleoptera (*Lepidiota mansueta, Odontotaenius disjunctus*), and Lepidoptera (*Cossus cossus*, *Diatraea saccharalis*)^30–34^.

The cellulose and hemicellulose fibers within plant cell walls are embedded in the pectin matrix, necessitating the initial digestion of pectin to provide substrates for the enzymes^35^. Research has demonstrated that the degradation of pectin could also rely on the activity of pectinase produced by insect gut bacteria. For instance, the gut symbiont *Stammera* of a leaf beetle *Cassida rubiginosa* possesses genes involved in pectin digestion^36^. Similarly, in insects that heavily rely on pollen as their primary nutrient source, accessing the nutrients within pollen requires overcoming the barrier of the pollen wall. The chemical composition of the pollen exine layers consists mainly of sporopolysin, while the inner layer contains pectin^37,38^. Metagenomic studies have revealed the presence of genes associated with pectin degradation in the gut bacteria *Gilliamella apicola* of honey bees, and in vitro culturing tests have confirmed its pectin degradation activity^39^. Lignin is another kind of common natural polymer with abundant content and complex structure that is difficult for insects to metabolize. Although fungi might be the main players for lignin degradation in insects, some gut bacteria have the ability to breakdown lignin^40^. Several studies have isolated and identified bacteria with lignin-degrading potential from the gut of termites^40–42^. Transcriptomic approaches also revealed that midgut microorganisms participate in lignin degradation in the longhorn beetle *Anoplophora glabripennis*^43,44^. These studies highlight the ability of various gut bacteria species to assist their hosts in the degradation of resilient plant polymers.

Meanwhile, a plant-based diet is often lacking in some of the nutrients necessary for herbivorous insect survival and development. To alleviate nutrient limitations, insects have evolved various gut bacteria-mediated strategies that allow them to take advantage of plants lacking certain nutrients^11^. Nitrogen is generally considered to be the limiting factor in the diet of herbivorous insects^45^. Apart from this, plant proteins lack essential amino acids (EAAs), so the growth and development of insects may be severely limited^46^. The gut bacteria of herbivorous insects can use atmospheric nitrogen sources for biological nitrogen fixation, such as the passalid beetle *Odontotaenius disjunctus* and the medfly C*eratitis capitata*^47,48^. In addition, herbivores insects’ gut bacteria employ nitrogenous waste recycling (NWR) as a key method to acquire supplemental nitrogen sources, utilizing these wastes to synthesize essential amino acids (EAAs) that can be reabsorbed by the insect hosts. For example, the symbiotic bacteria of the oriental fruit fly *B. dorsalis* can drive NWR with the help of *Morganella morganii* and *Klebsiella oxytoca*^49^. Similarly, gut bacteria have been found in leafcutter ants to recycle urea (and possibly uric acid) and use the recycled nitrogen to make large amounts of EAAs^50^. Besides, insects that feed on plant xylem and phloem sap have limited vitamin availability^51^. Gut bacteria have been shown to provide vitamins to insect hosts. Symbiotic bacteria colonizing the bean bug *Riptortus pedestris* midgut produce B vitamins that are scarce in the host insect’s soy diet^52^. Through genomic analysis, it was demonstrated that the gut bacteria (*Bartonellaceae*) of herbivorous ants (*Dolichoderus*) encode genes for several vitamins and all essential amino acid biosynthetic pathways^53^. The biosynthetic capability of vitamins exhibited strain-specific variability among different bacterial species. For example, while the *thiE* gene responsible for vitamin B1 synthesis was present in all members of the *Bartonellaceae* family, only one strain possessed a complete set of genes for the synthesis of vitamin B3.

### Gut bacteria degrade plant toxins

In addition to the aforementioned stubborn plant polymers that hinder insect feeding, herbivorous insect attacks can induce phytochemical resistance through the production of secondary metabolites, which often serve as defensive compounds to deter herbivores from feeding^54^. Aside from the insects’ inherent detoxifying metabolic ability, gut microbes significantly contribute to the degradation of the ingested phytoallelopathic substances^55–57^. For example, gut bacteria of the pine weevil (*Hylobius abietis*) can help weevils metabolize diterpene acids of Norway spruce, and the gut bacteria can even utilize diterpenoids as a carbon source and may produce nutrients to increase insect fitness^58^. For the primary pest of Chinese tea plants—*Curculio chinensis*, *Acinetobacter* species in the gut was identified to be involved in the degradation of tea saponin^59^. Some studies have found that caffeine is degraded in the gut of the coffee berry borer (*Hypothenemus hampei*), and have shown that *Pseudomonas* species play an important role in the degradation of caffeine^60^.

Furthermore, the gut microbiota exposed to challenging compounds, and defensive substances can dynamically adjust their structure or remain stable so as to better assist insects in adapting to such environmental stressors^61–63^. The viewpoint has been validated in research systems involving pine sawyers and bark beetles, among others. Gut-associated microbiota of the pine sawyer *Monochamus saltuarius* can shift in response to different dietary stimuli, which correlates with its diverse ability to metabolize secondary plant metabolites^64^. In contrast, the gut bacterial community of the bark beetle *D. valens* maintains resilience, enabling the beetle to catabolize pine defense chemicals^65^. Both pests are pine feeders, but the two present distinct and opposite patterns of gut microbiota changes, which remain unclear but are worth further investigation. The above examples collectively demonstrate that microorganisms in insect guts can aid in the rapid adaptation of phytophagous insects to plant secondary metabolites. Furthermore, insects utilize gut bacteria to degrade plant defense compounds, thereby allowing them to have more energy available for their own growth, development, and reproduction.

### Dysbiosis of gut microbiota is detrimental to insect survival

Plants have various defense mechanisms to resist herbivorous insects^66^, and the insect gut is frequently targeted by plant defenses. Both physical and chemical plant defenses can disrupt the protective peritrophic matrix (PM) of the gut, resulting in increased permeability and potential invasion by gut bacteria, leading to septicemia^67–69^. One study found that plant defenses against the fall armyworm *Spodoptera frugiperda* facilitated gut microbes to penetrate gut barriers, invade the body cavity, and worsen the negative impacts of plant defenses on the insect^70^. Similar observations were reported for the diamondback moth *Plutella xylostella*, where plant defensive substance (3E)-4,8-dimethyl-1,3,7-nonatriene (DMNT) disrupted midgut microbiota populations and PM, and midgut microbes helped plant toxins kill insects^13^. Even if the structure of the gut structure is not damaged, disturbance of the gut microbiota can still negatively affect insects. A recent study found that tomato plants colonized by *Trichoderma* negatively affected the development and survival of another lepidopteran pest *Spodoptera littoralis* by altering the structure and function of the gut microbiota of *S. littoralis* larvae^71^.

Consequently, apart from the beneficial effects of gut bacteria, certain gut bacteria can turn pathogenic when insects are exposed to plant defenses^64^. The composition and structure of the gut microbiota are crucial for the functional attributes of each gut bacterium. Hence, we are compelled to contemplate whether the gut microbiota influenced by plant defense impacts insect development or if alterations in insect physiology induced by plant defense lead to subsequent modifications in the gut microbiota, thereby further influencing the physiological processes of the insect. Addressing the former hypothesis could be achieved through direct transplantation of the plant-altered microbiota into axenic insects under conditions without plant defense. However, investigating the latter scenario poses more intricate challenges in research design and interpretation. Overall, gut bacteria primarily act as facilitators for insects when the plant’s defense is relatively weak. However, in situations where plants exhibit strong defenses or when insects are in poor physiological condition, gut bacteria often transform into pathogenic bacteria and accelerate the mortality of the pests, as supported by the examples we described above.

## Gut bacteria influence the insect–plant relationship by impacting plant physiology

**Fig. 2:**
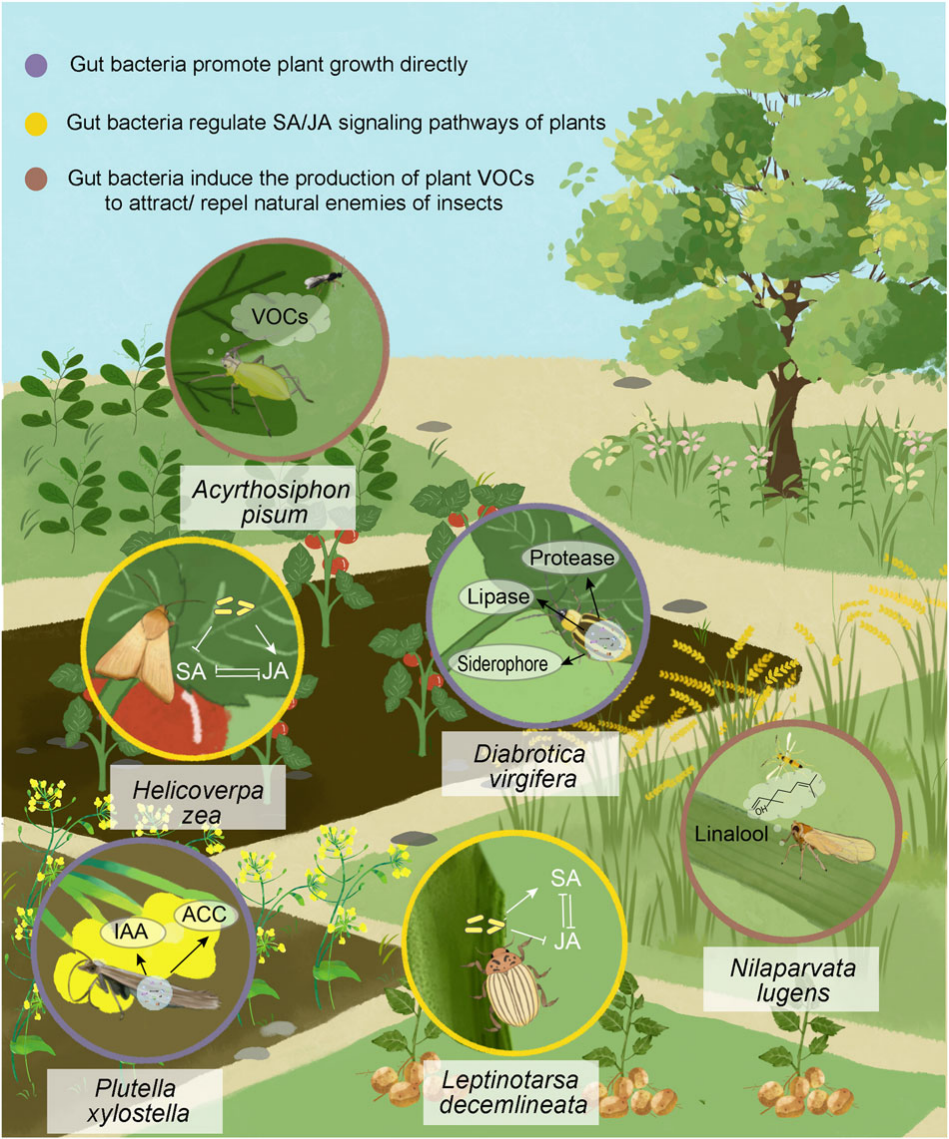
Gut bacteria participate in plant–insect interactions. The purple circle represents an example of gut bacteria promoting plant growth directly. The yellow one represents an example of gut bacteria regulating SA/JA signaling pathways of plants. And the brown circle shows an example of gut bacteria inducing the production of plant VOCs to attract/repel natural enemies of insects.

Gut microbiota not only impact the interaction between insects and plants by means of direct effects on the former, but they also shape this association by exerting an influence on the latter (Fig. 2). Insects are able to transmit their gut bacteria to plants through various means such as saliva, reflux, feces, eggs, and honeydew^72–74^, which significantly influence the plants’ physiology in diverse ways. Firstly, some gut bacteria can act as promoters of plant growth like plant-associated microbes have shown. Furthermore, gut bacteria affect the adaptability of insects and plants by inducing plant defenses, including direct defenses mediated by plant hormones, and indirect defenses that attract natural enemies through volatile compounds.

### Gut bacteria promote plant growth directly

Plants harbor a myriad of bacteria, including those capable of promoting plant growth^75–77^. Meanwhile, herbivorous insects constantly feed, lay eggs, and traverse plants, providing ample opportunities for plant-associated bacteria to colonize their guts^78^. The colonization of plant leaf-associated bacteria in the gut of leaf-eating insects is logical and comprehensible, exemplified by the ability of the poplar-associated bacterium *Pseudomonas putida* to colonize the gut of the leaf beetle *Plagiodera versicolora*^79^. Even soil bacteria can be transmitted to aboveground insects (foliar-feeding insects) and colonize their gut^78^.

Moreover, nutrient cycling rates in the insect gut environment are much higher than in soil and plants, leading to a greater potential for disturbance. Consequently, insect gut bacteria are more metabolically versatile than bacteria found in other environments^80^, providing a basis for them to influence plant functions. For example, *Pseudomonas putida*, the gut bacterium of *P. versicolora*, benefits the host plant by promoting growth and reducing trichloroethane phytotoxicity^81^. Previous research has demonstrated that eight gut bacteria of diamondback moth larvae (*P. xylostella*) possess plant growth-promoting traits, including those that fix atmospheric nitrogen, produce indole acetic acid (IAA) and salicylic acid (SA), solubilize phosphates, promote zinc absorption, and produce glucanases, chitinases and 1-aminocyclopropane-1-carboxylate (ACC) deaminase^80^. Another study found that gut bacteria in *Diabrotica virgifera* can promote the growth of tomato plants^82^. Based on the reasons mentioned above and these examples, we have sufficient reasons to believe that more and more discoveries of insect gut bacteria that affect host plant growth will be reported.

### Gut bacteria regulate SA/JA signaling pathways of plants

Plant defense is triggered when the plant detects various insect-derived cues, such as tissue damage, oral secretions, oviposition, and frass, and is mediated by phytohormones to elicit resistance^83,84^. In response to chewing insects, plants activate jasmonic acid (JA)-mediated defenses, while biotrophic pathogens and some piercing–sucking insects elicit salicylic acid (SA)-mediated defenses^85,86^. The antagonistic relationship between JA and SA signaling pathways enables plants to fine-tune their defense responses to specific organisms^87,88^.

Herbivorous insect gut bacteria can alter the JA/SA pathways and inhibit plant defense by secreting gut bacteria from oral secretions and frass onto plants during feeding. For example, the Colorado potato beetle (*Leptinotarsa decemlineata*) carried gut endosymbiotic bacteria that were deposited on plants through the beetle’s oral secretions, and the authors have shown that associated bacteria enhanced the SA signaling pathway and suppressed JA-mediated defenses in tomato^89^. Similarly, oral secretion-associated bacteria of the cotton leafworm *Spodoptera litura* contributed to the ability of host insects to manipulate plant defenses by promoting the *Arabidopsis* SA signaling pathway and inhibiting the JA signaling pathway^90^. Nevertheless, in some cases, insect gut bacteria can induce the expression of defense-related enzymes and JA regulatory genes. For instance, the gut bacteria of the corn earworm *Helicoverpa zea* (*Enterobacter ludwigii*) induced the expression of polyphenol oxidase and JA regulatory genes, while inhibiting SA disease-related genes, providing potential benefit to the tomato plant’s fitness^74^.

The current research on gut bacteria that can influence JA and SA pathways is mostly focused on chewing insects, while there are fewer studies on the gut bacteria of piercing–sucking insects. Overall, the interaction between insect gut bacteria and plant SA/JA signaling is complex and can involve both synergistic and antagonistic effects, depending on the specific insect species and bacterial strains involved, as well as the host plant^57,91^. Furthermore, on one hand, gut bacteria of herbivorous insects can promote plant growth, while on the other hand, they can also modulate plant defense responses through JA or SA signaling pathways. In fact, a trade-off exists between plant defense and growth^81^. Therefore, it is worth exploring how plants balance these seemingly contradictory signals derived from gut bacteria.

### Gut bacteria induce the production of plant VOCs to attract/ repel natural enemies of insects

Plants respond to herbivore attacks by emitting specific blends of herbivore-induced plant volatiles (HIPVs), which can not only directly affect the herbivores but also indirectly impact them by attracting their natural enemies^92,93^. Without surprise, the induction of the indirect herbivore-defense mechanism can be affected by insect gut bacteria. For instance, bacteria present in the honeydew of the rice brown planthopper (*Nilaparvata lugens*; BPH) strongly elicit indirect defenses in rice, and the release of volatile organic compounds from the leaves serve as attractants for natural enemies of the herbivores. Almost all of the microbes isolated from the honeydew were previously reported to be gut symbiotic bacteria of BPH^72^. On the contrary, the bacterial symbiont *Hamiltonella defensa* has been shown to reduce the recruitment of parasitic wasps and improve pea aphid (*Acyrthosiphon pisum*) fitness by reducing the amount of volatile compounds produced by plants^94^.

It seems that whether the gut bacteria-stimulated chemical compounds attract or repel natural enemies depends on the insect, natural enemy, and plant species involved, and this complex interaction mediated by gut bacteria is worth further investigation. Regardless of whether the gut-bacteria-mediated HIPVs attract or repel natural enemies, we should carefully consider the ecological significance of gut bacteria possessing these functions in further research.

## Gut bacteria influence the insect–plant relationship by modulating the third trophic level species within the ecological network

**Fig. 3:**
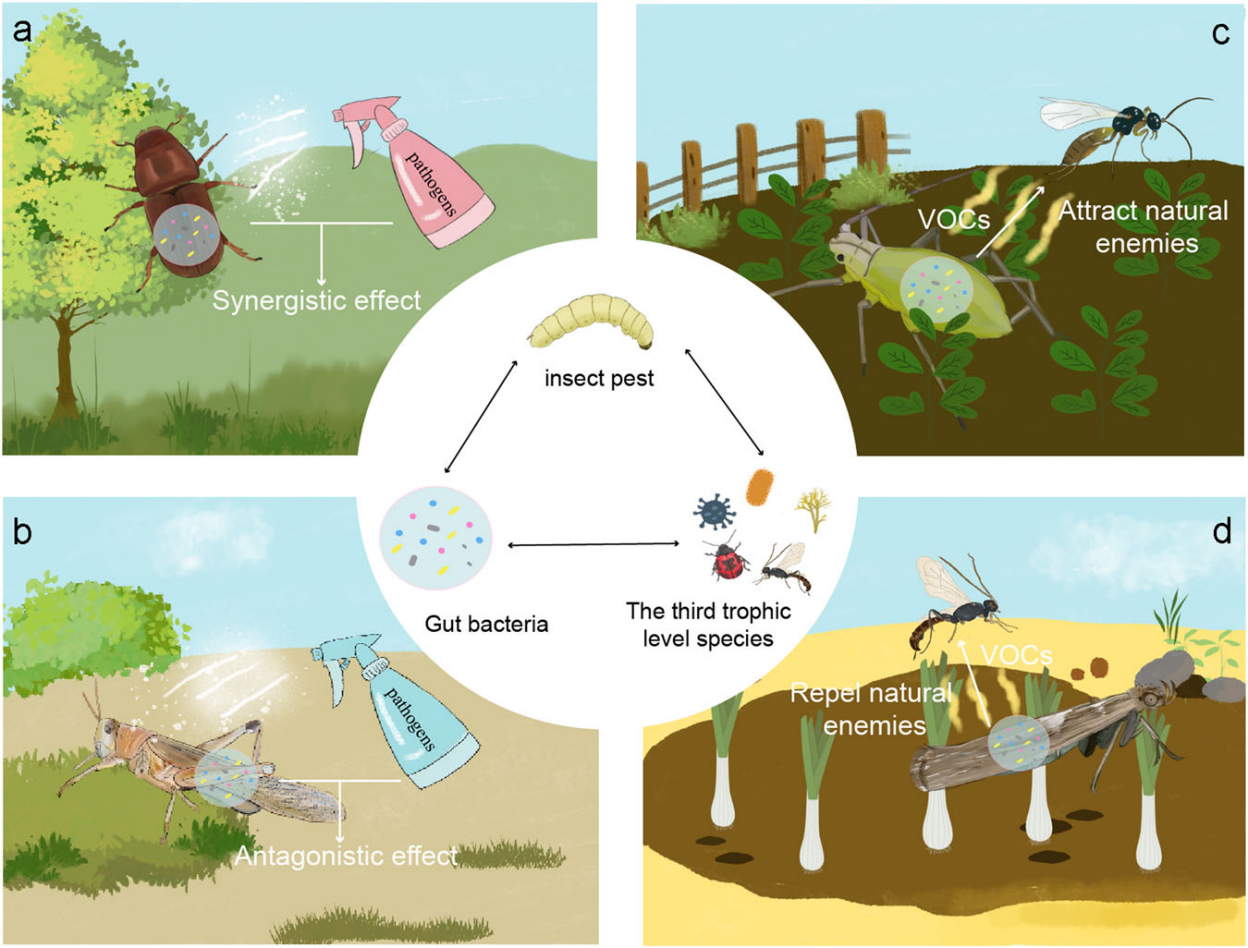
Gut bacteria participate in plant–insect interactions by exerting an influence on the third trophic-level species. **a** Some gut bacteria show synergistic effects with entomopathogens to kill insects; **b** gut bacteria in several insects possess antagonistic effects against pathogens; **c** the VOCs produced by insects’ gut bacteria can attract natural enemies; **d** VOCs produced by gut bacteria of insects may also repel their natural enemies.

As mentioned earlier, the insect–plant interaction process involves the participation of other organisms from different trophic levels, either through gut bacteria-mediated HIPVs or through intentional interventions, or other means. Interestingly, the involvement of organisms from another trophic level can alter the existing pairwise interactions between insects and plants (Fig. 3). For example, when pathogenic microorganisms are employed for insect pest control, gut bacteria may play a synergistic or antagonistic role in influencing the virulence of the microbial insecticide. Additionally, different gut microbiota can stimulate plants to produce varied HIPVs and further elicit varying behavioral responses in insect natural enemies, thereby altering the insect–plant interaction dynamics.

### Gut bacteria alter the virulence of microbial insecticides

Microbial insecticides, which typically consist of entomopathogenic bacteria, fungi, or viruses^95^, have developed various pathogenic factors and toxins in the evolutionary arms race with host insects, making them powerful weapons to control insect pests. However, the pathogenicity of these pathogenic microorganisms can be easily influenced by a range of biological factors. Among them, gut bacteria often become involved in the process by promoting or inhibiting the infection. For instance, a previous study discovered that *Beauveria bassiana* infection in bark beetle *D. valens* LeConte caused dysbiosis of gut microbiota and overgrowth of the bacterium *Erwinia* sp. in the gut, thereby accelerating beetle mortality^12^. Another study showed that the Cry1Ac protoxin produced by *Bacillus thuringiensis* (Bt) induced a dynamic change in the midgut microbiota of the diamondback moth *P. xylostella*, and that the Bt Cry1Ac protoxin interacts with the gut microbiota to accelerate the mortality of larvae^96^. In contrast, the loss of gut microbiota significantly decreased the Bt susceptibility of *P. xylostella* larvae^96^. Similar insecticidal mechanisms of synergistic action between pathogenic microorganisms and gut bacteria were also reported in the European gypsy moth (*Lymantria dispar asiatica*) and the tobacco hornworm (*Manduca sexta*)^97,98^.

Apart from the synergistic effect between pathogens and insect associated bacteria interactions, antagonistic effects between these two agents were also widely reported. Following *Nosema bombycis* infection, the abundance of *Enterococcus* in the silkworm *Bombyx mori*’s gut increased, and *Enterococcus faecalis LX10* reduced the *N. bombycis* spore germination rate and the infection efficiency in vitro and in vivo^99^. In the spruce beetle, *D. rufipennis* bacteria present in oral secretions inhibited the growth of entomopathogenic fungi^100^. Similarly, gut bacteria of the locust *Schistocerca gregaria* and Scarabs (*Holotrichia oblita*, *Holotrichia parallela*, and *Anomala corpulenta*) have also been demonstrated to exert antimicrobial activity similar to the above study^101,102^. Indeed, the interactions between pathogenic microorganisms and gut bacteria do not adhere to a consistent pattern of synergy or antagonism. Even the same bacterial species, such as *Enterococcus faecalis*, can exert completely opposite effects when confronted with different pathogens within various host insects. A better understanding of pathogenic microorganisms–insect–gut bacteria interactions is critical for the development of more effective microbial insecticide.

### Gut bacteria produce VOCs to attract or repel natural enemies of the host

Insect natural enemies, as another important trophic level similar to entomopathogens, can also intervene and alter the insect–plant relationships^103^. Natural enemies of herbivorous insects, including predators and parasitoids, typically locate their concealed prey in structurally complex environments using volatile chemical cues^104^. In most cases, the behavior of natural enemies is mediated by plant or host volatiles, which can either repel or attract them. Studies have shown that volatile compounds produced by gut bacteria in insects can also affect the behavior of natural enemies. For instance, researchers have demonstrated that volatile compounds produced by bacteria in an aphid (*Acyrthosiphon pisum*) honeydew can attract predators and certain chemicals produced by *Staphylococcus sciuri* have been identified as attractants and ovipositional stimulants for the predator hoverfly (*Episyrphus balteatus*)^14^. However, other studies have shown a negative relationship between honeydew bacteria and the attraction of the aphid parasitoid *Aphidius colemani*^105^. Additionally, Thibout et al. found that the volatiles used by the parasitoid *Diadromus pulchellus* to locate its host, the leek moth (*Acrolepiopsis assectella*), are produced by the bacteria developing in the frass of larvae^106^.

Insect gut bacteria often serve as a reservoir, being present in the honeydew and feces of the insects through excretion, feeding, and other activities. The aforementioned bacteria, which have the ability to influence natural enemies, are closely related to the gut bacteria and can be broadly classified as such. Moreover, as previously discussed, gut bacteria can produce volatile compounds that influence various behaviors of insects, including feeding and oviposition. It is plausible to consider that over the course of long-term evolution, insect natural enemies may have acquired the ability to perceive and interpret these “chemical messages” to locate and target their prey. Therefore, besides exploring whether natural enemies can decode the chemical information produced by insect gut bacteria, we should also pay attention to how insects fine-tune their gut microbiota to prevent natural enemies from deciphering this information.

## The leverage of using gut bacteria for pest management

**Fig. 4:**
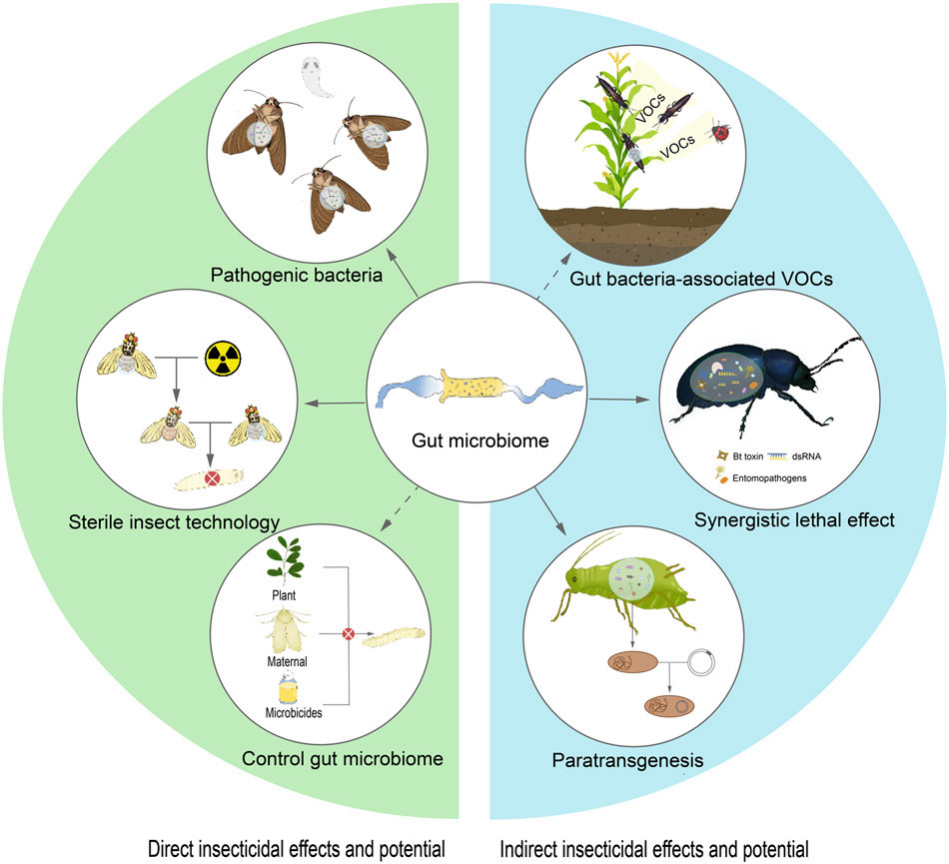
The vast potential of gut microbiota for pest management.

Entomopathogenic bacteria and their toxins have been successfully developed and utilized against a wide range of pests. However, the susceptibility of insect pests to microbial insecticides varies, and some pests have developed resistance to pathogenic bacteria^107–109^, necessitating the development of more active insecticidal bacteria. In this context, gut bacteria have received increasing attention as potential sources of insecticidal bacteria (Fig. 4 and Table 2). Besides, with the advancement of modern molecular biology techniques, microbial control technology has expanded beyond the use of a single bacteria, with RNAi, sterile insect technology, and paratransgenesis integrated with gut bacteria for pest control (Fig. 4 and Table 2).

**Table 2:** Direct and indirect insecticidal effects and potential of insect gut bacteria.

Insect	Order	Gut bacteria	Application	References
**1. Direct insecticidal effects and potential of gut bacteria**				
*Manduca sexta*	Lepidoptera	*Enterococcus faecalis*	Direct insecticidal effect	^98^
*Spodoptera litura*	Lepidoptera	*Serratia marcescens* *Enterobacter cloacae*	Direct insecticidal effect	^112,161,162^
*Hylesia metabus*	Lepidoptera	*Bacillus licheniformis* *Planococcus* sp. Other 4 gut bacterial species	Direct insecticidal effect	^113^
*Curculio dieckmanni*	Coleoptera	*Serratia marcescens*	Direct insecticidal effect	^163^
*Agrypnus murinus*	Coleoptera	*Pseudomonas protegens*	Direct insecticidal effect	^164^
*Schistocerca gregaria*	Orthoptera	*Bacillus weihenstephanensis Pseudomonas* sp.	Direct insecticidal effect	^165^
*Adelphocoris suturalis*	Hemiptera	*Serratia marcescens*	Direct insecticidal effect	^166^
*Ceratitis capitata*	Diptera	*Providencia rettgeri*	Direct insecticidal effect	^167^
*Ceratitis capitata*	Diptera	*Klebiella xytoca* *Enterobacter* sp.	The sterile insect technique	^118,168^
*Bactrocera dorsalis*	Diptera	*Morganellamorganii Moellerella wisconsensis*	The sterile insect technique	^169^
**2. Indirect insecticidal effects and potential of gut bacteria**				
*Dendroctonus valens*	Coleoptera	*Lactococcus lactis* *Rhodococcus* sp. Other 11 gut bacterial species	Synthesize VOCs	^123,124^
*Lymantria dispar*	Lepidoptera	Gut microbiota	Synergy with entomopathogens	^97^
*Spodoptera exigua*	Lepidoptera	Gut microbiota	Synergy with entomopathogens	^170^
*Plagiodera versicolora*	Coleoptera	*Pseudomonas putida*	Synergy with lethal dsRNA/Bt	^79,171^
*Rhodnius prolixus*	Hemiptera	*Rhodococcus rhodnii*	Deliver lethal dsRNAs	^128^
*Acyrthosiphon pisum*	Hemiptera	*Serratia symbiotica*	Deliver lethal dsRNAs	^172^
*Aphis fabae*	Hemiptera	*Serratia symbiotica*	Paratransgenesis	^136^

### Direct insecticidal effects and potential of gut bacteria

Gut bacteria become virulent under specific physiological or environmental conditions or due to disruption of normal microbial composition^71,79,110^. Two main mechanisms of insect-killing by gut bacteria include toxin-induced starvation and sepsis caused by the microbiome^111^. *Enterobacter cloacae*, for example, can induce pathogenicity in its host the cotton leafworm *S. litura* by causing starvation and disrupting the normal gut microbiota when fed orally^112^. In another instance, the presence of *Enterococcus faecalis* in the midgut of the tobacco hornworm *M. sexta* larvae does not cause obvious disease, but it dies quickly when injected directly into the larval hemolymph^98^. Other pathogenic gut bacteria include *Serratia marcescens*, *Bacillus licheniformis, Pseudomonas aeruginosa, Proteus vulgaris, Alcaligenes faecalis*, and *Planococcus* sp.^113^. As all of these bacteria have the potential to kill pests, they can be considered as potential biological control agents for insects in pest management.

Sterile insect technology (SIT) involves mass-rearing and δ-irradiation sterilization of male insects, which are then released into the target area to compete with wild males for mating with wild females, and has been successfully used in pest control of Trypetidae^114–117^. Surprisingly, studies have shown that the mating competitiveness of sterile medfly male (*C. capitata*) obtained by radiation treatment in the field was significantly reduced, with a significant decrease in the abundance of *Klebiella* in the male gut and an increase in the conditional pathogenic *Pseudomonas*^118^. Feeding *K. xytoca* was able to enhance the mating competitiveness of infertile males^118^. Therefore, by manipulating the gut microbiota through certain methods (e.g., increasing *Klebsiella* or reducing *Pseudomonas* abundance), we may improve the competitive ability of sterile medfly males, thus leading to better pest control.

Collectively, given the direct role of gut bacteria in enhancing the adaptability of insects^119–121^, manipulating the insect gut microbiome has the potential to either increase or decrease insect fitness. We can utilize this knowledge for pest control purposes. Previous research has been conducted to leverage plant-mediated expression of antimicrobial peptides as a potential strategy to regulate plant-associated microbiota^122^. This method holds promise for regulating the gut microbiota of herbivorous insects, wherein plants could be engineered to express antimicrobial compounds or facilitate the transmission of beneficial microorganisms based on pest control requirements. However, further comprehensive studies are necessary to assess their efficacy and safety in practical applications.

### Indirect insecticidal effects and potential of gut bacteria

Apart from directly influencing the fitness of herbivorous, insect gut bacteria can also produce or induce plant VOCs to attract other pests or natural enemies^10,14,72^. However, despite their potential, few examples of using VOCs associated with gut bacteria for pest control currently exist, e.g., while the protective effects of the multifunctional pheromone (verbenone) on *Pinus contorta* trees from the bark beetle *D. valens* have been well-documented, the discovery of its synthesis by gut bacteria came at a later stage^123,124^. We emphasize the consideration of VOCs associated with gut microbes in integrated pest management strategies, based on two primary reasons. Firstly, research in this area has the potential to reveal novel VOCs for pest management. Secondly, it provides an opportunity to utilize insect gut bacteria as “fermentation factories” to produce these VOCs.

Symbiont-mediated RNAi (SMR), which involves genetically engineering gut symbionts to continuously produce and deliver dsRNA within pests, has been successfully applied to a variety of pests^125–127^. For example, Taracena et al. genetically modified the symbiotic bacterium (*Rhodococcus rhodnii*) of a blood-sucking bug *Rhodnius prolixus* to express dsRNA targeting antioxidant function, resulting in a reduction in its oviposition rate^128^. Similar examples of modifying insect gut bacteria have also been reported in honeybees^129^. As orally ingested dsRNA must pass through the gut and enter epithelial cells and/or hemolymph to function, there is a high likelihood of interaction between gut bacteria and dsRNA. This hypothesis was confirmed in a study on a leaf beetle *P. versicolora*. Ingestion of dsRNA by *P. versicolora* led to the disturbance of gut microbiota, and the degradation products of dsRNA by insects preferentially promoted the growth of insect pathogenic bacteria, thereby increasing the insecticidal efficiency of RNAi^79^. These results indicate that it is the degradation products of dsRNA, rather than the knock-down of the targeted gene, that influenced the gut microbiota. Moreover, the altered microbiota subsequently led to different RNAi effects. Although there are currently limited studies on the combination of gut bacteria and RNAi for pest management, this provides a promising approach for developing pest control mechanisms based on gut bacteria.

The fields of synthetic biology and symbiotic insect bacteria have merged to create a new pest control strategy called paratransgenesis. This technique involves using symbiotic bacteria as gene expression vectors to introduce target genes into insects^130^. The expression of foreign genes interferes with pathogen development or insect fitness traits (e.g., proteotoxoids) for pest control purposes^131,132^. Unlike host-dependent endosymbiosis, gut bacteria are often culturable and easier to manipulate genetically and therefore more suitable as transgenic vectors. They can be easily reintroduced to host insects by oral ingestion and spread through the environment by horizontal transfer^9^. Another advantage of using gut bacteria as transgenic vectors is that they have the ability to colonize a variety of different insects, and can be passed horizontally and vertically from one generation to another^133^. For example, *Serratia* sp. (AS1) and *Pantoea agglomerans*, which are present in the gut of *Anopheles* mosquitoes, have been genetically engineered to secrete anti-Plasmodium effector protein and feeding the recombinant strain can inhibit the development of *Plasmodium falciparum* in mosquitoes^134,135^. Recently, the feasibility of paratransgenesis has also been demonstrated in agricultural pests. *Serratia symbiotica* CWBI-2.3 T, a culturable enteric-associated bacterium isolated from the black bean aphid and which can be genetically engineered^136^. Although paratransgenesis has not been extensively used in agriculture and forestry pest control, it holds promise as a potential approach for developing more scientifically based pest control methods.

## Future perspectives and challenges

Here, we discuss the diverse functions of gut bacteria in the context of insect–plant interactions, which includes altering insect adaptability to host plants, influencing insects’ perference on different plants, regulating plant growth and defense, changing pathogenic virulence of microorganisms, and attracting/repelling natural enemies. Thus, gut bacteria is crucial for the development of novel tools in pest control methods. With advances in RNAi and paratransgenesis, the use of gut bacteria for pest control has become more diverse. Moreover, compared to chemical pesticides, biological control agents are less likely to cause environmental pollution. Therefore, rational utilization of these multispecies cascading interactions can be considered as an eco-friendly and novel approach for pest control.

Despite the promising potential of using gut bacteria for pest control, significant challenges still exist in their application. Firstly, our understanding of the role of gut bacteria in insect–plant interactions and behind mechanisms is currently incomplete due to the complexity of these interactions. Obtaining axenic insects is a major challenge in studying these interactions, as only a small fraction of agricultural and forestry pests have been successfully reared under axenic conditions. Axenic rearing approaches allow for the deconstruction and reconstruction of insect–plant–microbe interactions to identify the functions of specific gut bacteria in insect pest–plant interactions^137,138^. Secondly, the vast majority of gut bacteria are unculturable, which makes functional and practical studies difficult. Although recent advances in large-scale culturing methods, particularly culturomics, have made it possible to culture some gut bacteria, the challenge of large-scale cultivation and application remains^139,140^.Thirdly, in addition to gut bacteria, insect–plant interaction in the field often involves multiple other species, and conclusions based on laboratory studies may be varied when applied in real-world conditions, particularly with the addition of abiotic factors such as climate change. Therefore, further research is needed to integrate the specific mechanisms by which insect, plant, and gut microbial communities interact under different climatic and ecological variables. This is undoubtedly a significant challenge for researchers.

Additionally, when considering the use of other biological technologies such as RNAi or SIT in conjunction with gut bacteria or targeting the pest–gut bacteria interaction for pest control, it is necessary to take into account the efficiency and cost of these techniques^51^. Similar to the bottleneck encountered in developing RNAi-based insect pest control technologies, gut bacteria, and RNAi-based insect control technology also require consideration of insect uptake, degradation of dsRNA, and mode of delivery^141,142^. Furthermore, the large-scale production of dsRNA and its application in the field is a cumbersome and expensive process^143^. Similarly, SIT technology requires a substantial population of males to compete with wild males, making field application difficult and costly^144^.

Despite these challenges, recent advances in macrogenomics and transcriptomics have greatly increased our understanding of the functions of gut bacteria in insect–plant interactions^145^, providing a theoretical basis for the development of new pest management strategies. In the face of rampant pest resistance to chemical pesticides, Bt toxin, and dsRNA, it is strategically important to innovate and develop new pest management strategies that target the symbiotic relationship between pests and their gut microbiota, or gut bacteria-based pest management. Therefore, further research on the molecular mechanisms of gut bacteria in insect–plant interactions is not only of great scientific value but also has far-reaching practical implications.

### Supplementary information

nr-reporting-summary
